# Lentiviral Gag Assembly Analyzed through the Functional Characterization of Chimeric Simian Immunodeficiency Viruses Expressing Different Domains of the Feline Immunodeficiency Virus Capsid Protein

**DOI:** 10.1371/journal.pone.0114299

**Published:** 2014-12-02

**Authors:** María J. Esteva, José L. Affranchino, Silvia A. González

**Affiliations:** Laboratorio de Virología, Universidad de Belgrano (UB) and Consejo Nacional de Investigaciones Científicas y Técnicas (CONICET), Buenos Aires, Argentina; University of Pittsburgh Center for Vaccine Research, United States of America

## Abstract

To gain insight into the functional relationship between the capsid (CA) domains of the Gag polyproteins of simian and feline immunodeficiency viruses (SIV and FIV, respectively), we constructed chimeric SIVs in which the CA-coding region was partially or totally replaced by the equivalent region of the FIV CA. The phenotypic characterization of the chimeras allowed us to group them into three categories: the chimeric viruses that, while being assembly-competent, exhibit a virion-associated unstable FIV CA; a second group represented only by the chimeric SIV carrying the N-terminal domain (NTD) of the FIV CA which proved to be assembly-defective; and a third group constituted by the chimeric viruses that produce virions exhibiting a mature and stable FIV CA protein, and which incorporate the envelope glycoprotein and contain wild-type levels of viral genome RNA and reverse transcriptase. Further analysis of the latter group of chimeric SIVs demonstrated that they are non-infectious due to a post-entry impairment, such as uncoating of the viral core, reverse transcription or nuclear import of the preintegration complex. Furthermore, we show here that the carboxyl-terminus domain (CTD) of the FIV CA has an intrinsic ability to dimerize *in vitro* and form high-molecular-weight oligomers, which, together with our finding that the FIV CA-CTD is sufficient to confer assembly competence to the resulting chimeric SIV Gag polyprotein, provides evidence that the CA-CTD exhibits more functional plasticity than the CA-NTD. Taken together, our results provide relevant information on the biological relationship between the CA proteins of primate and nonprimate lentiviruses.

## Introduction

Virion morphogenesis in lentiviruses is the result of a series of steps driven by multimerization of the structural polyprotein Gag at the plasma membrane of the infected cell (reviewed in refs. [1], [2]). Indeed, the intrinsic biological property of Gag to self-assemble into spherical virus-like particles both in cell cultures or in *in vitro* systems is well documented [3]–[9]. Like in all retroviruses, the simian immunodeficiency virus (SIV) Gag precursor is composed of the three functionally conserved domains: matrix (MA), which not only contains the molecular determinants necessary for Gag targeting and association with the plasma membrane but also participates in envelope (Env) glycoprotein incorporation into virions [10]–[12], capsid (CA) which in the mature virion constitutes the protein shell enclosing the dimeric RNA genome, and nucleocapsid (NC), which is involved in genomic RNA packaging and reverse transcription [1], [2]. SIV Gag also contains the C-terminal p6 domain which bears binding sites for the accessory viral proteins Vpr and Vpx (in some SIVs) [13] as well as for components of the endosomal sorting complexes required for transport (ESCRT) implicated in virus budding (reviewed in refs. [2], [14]). In addition, the SIV precursor contains two short spacer peptides SP1 and SP2 which separate the CA and NC and the NC and p6 domains, respectively.

Concomitantly with virus budding from the host cell, the Gag precursor is cleaved by the virus-encoded protease into its functional domains [15]. This step is accompanied by a series of structural rearrangements that convert the roughly spherical Gag shell of the immature virion into the mature infectious particle exhibiting the characteristic lentiviral electron-dense conical core [1], [2], [16]. In this regard, the central CA domain of Gag plays distinct roles during lentiviral morphogenesis: as part of the Gag precursor, it participates in the protein-protein interactions that drive Gag multimerization into immature particles [9], [17]–[21], whereas as an independent protein of the mature virion that self-assembles into the core structure, it protects the viral components required for the next steps of virus infection and spreading [2], [17], [22], [23]. The CA domains of retroviral Gag polyproteins exhibit low sequence similarity except for a 20-amino-acid motif known as the major homology region (MHR) which is unique in that it is conserved across retroviruses [24]. However, the comparison of the solution structures of different retroviral CA proteins shows a common organization in two highly α-helical regions that fold independently of each other: an N-terminal domain (CA-NTD) that is linked via a flexible region to a C-terminal domain (CA-CTD) [1], [2], [25], [26]. Indeed, it has been shown for different orthoretroviruses that the assembly of Gag into particles results in the formation of a hexagonal lattice in which the CA-NTD organizes into hexameric rings connected by CA-CTD homodimers [18], [19], [21], [26]–[28].

Most of the work on lentiviral CA proteins has almost exclusively focused on that of HIV-1. In this regard, numerous structural studies have compared the architecture of the immature HIV-1 Gag particle [18], [20], [21] with that of the mature virion exhibiting the CA-made core [26], [28]–[33]. These studies have provided a detailed model of the intra- and inter-hexameric subunit interactions that are established between the CA-NTD and CA-CTD upon HIV-1 Gag assembly. Moreover, a wealth of biochemical experiments have helped to define the contributions of the HIV-1 CA to the multifunctional properties of Gag during the virus life cycle such as its role in virion uncoating and nuclear import of the preintegration complex [17], [23], [34]–[38] as well as to identify the cellular proteins that are able to block virus replication by interacting either directly or indirectly with the CA protein [36], [39]–[42].

By contrast, little is known about other lentiviral CA proteins. As retroviruses belonging to the lentivirus genus, SIV and the feline immunodeficiency viruses (FIV) have common structural and biological properties but also exhibit important differences, which reflects both their evolutionary relationship and divergence. These lentiviruses share the same cell tropism (CD4+ T lymphocytes and macrophages) but utilize different receptor/coreceptor complexes to enter their target cells (CD4 and CCR5 for SIV; CD134 and CXCR4 for FIV) [43]–[47]. Moreover, the FIV genome lacks *tat*, *vpr* and *nef* which are iconic genes of primate lentiviruses [48] and *vpx* which is present only in some SIVs and HIV-2 [49]–[51]. Instead, FIV codes for the multifunctional protein Orf-A/Orf-2 which is involved in virus production and infectivity and has been shown to be related to the primate lentiviral protein Vpr [52], [53]. Moreover, FIV Gag contains the C-terminal p2 peptide, an 18-amino-acid-long domain that harbors the highly conserved PSAP motif involved in promoting virion budding, which is functionally equivalent to the p6 region in the Gag polyproteins of HIV and SIV [54]–[56].

With regard to the CA domains of the SIV and FIV Gag polyproteins, we have previously demonstrated that the C-terminal third portion of the CA and the entire NC is the minimal SIV Gag subdomain capable of interacting *in vitro* with GagΔp6 at wild-type levels [9]. Furthermore, experiments performed with FIV Gag have allowed us to show that the CA-NC region is the principal Gag domain responsible for the protein-protein interactions that drive immature particle assembly [57].

Therefore, to gain insight into the functional equivalence between the SIV and FIV CA proteins and to investigate whether the structurally distinct regions in the CA domain of SIV Gag can be functionally replaced by their cognate FIV CA counterparts, we characterized the assembly and infectivity phenotypes of chimeric SIVs carrying different FIV CA-derived regions.

## Results

### Construction of the chimeric SIV proviral DNAs

**Figure 1:**
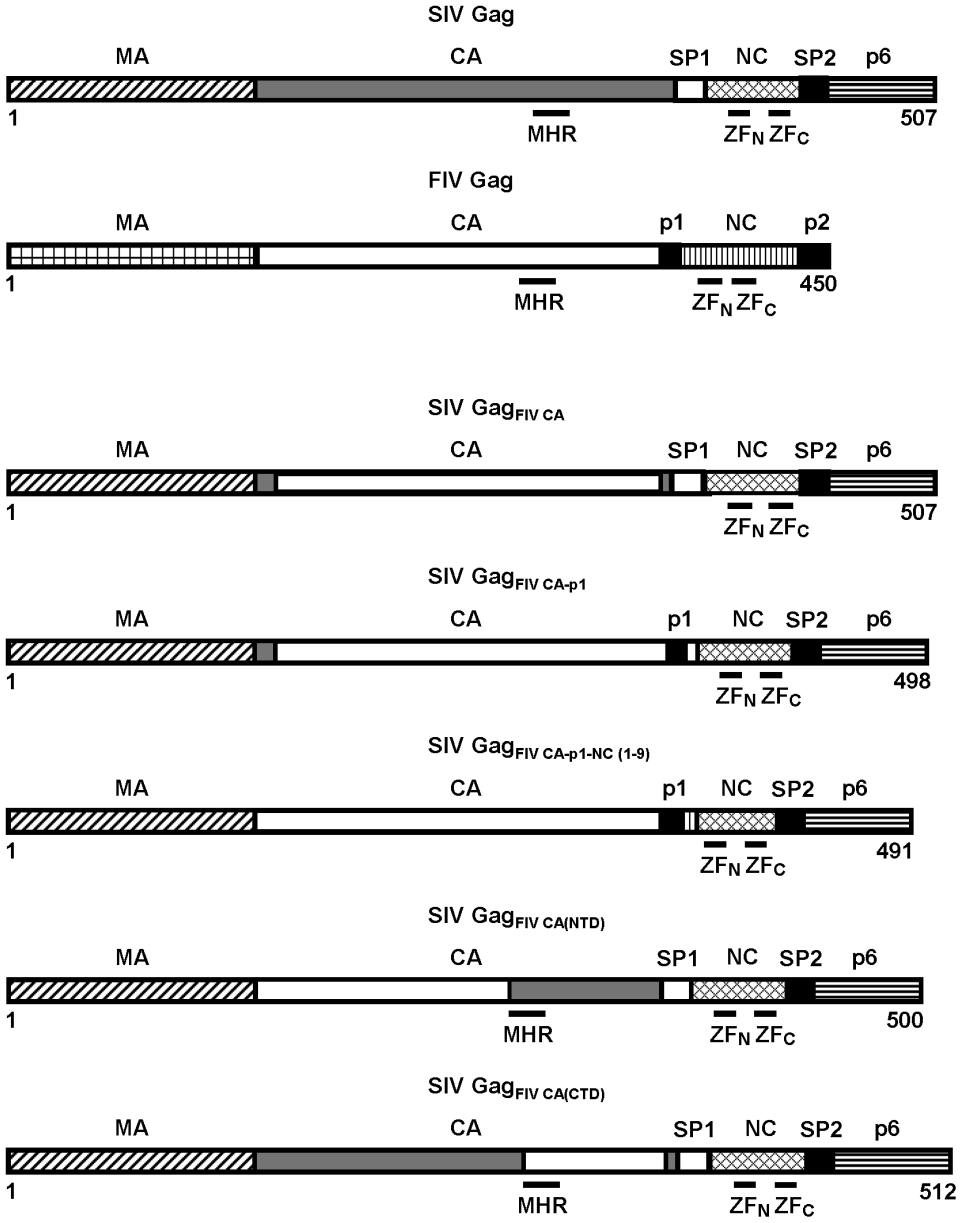
Schematic diagram of the chimeric Gag polyproteins encoded by the proviral SIV constructs. The organization of the wild-type SIV and FIV Gag precursors is depicted at the top showing the structurally conserved domains (MA, CA, and NC), the C-terminal p6 domain in SIV Gag, as well as the spacer peptides (SP1 and SP2 in SIV Gag; p1 and p2 in FIV Gag). The positions of the CA MHR, and the N-terminal (ZFN) and C-terminal (ZFC) zinc-finger motifs in the NC domains of both SIV and FIV Gag proteins are indicated: The numbers refer to the length of each of the chimeric Gag polyproteins with residue 1 corresponding to the initiator methionine in the Gag precursors.

The analysis of the SIV and FIV CA primary sequences reveals a similar organization: a CA-NTD of 151 residues for SIV and of 144 amino acids for FIV, and a CA-CTD that includes the MHR and whose length is 79 and 78 residues for SIV and FIV, respectively. However, the SIV and FIV CA proteins only share 30% identity and 52% similarity at the amino acid level. Therefore, to investigate the functional relationship between the CA domains of these two phylogenetically distant lentiviruses, we generated a series of SIV_SMM-PBj_ proviral DNAs carrying different CA-coding sequences of the Petaluma isolate of FIV (Fig. 1). In the chimeric virus SIV_FIV CA_ the first thirteen SIV CA residues were joined to the FIV CA sequences coding for amino acids 14–222 without eliminating the C-terminal six residues of the SIV CA so as to ensure proper processing at both the MA-CA and CA-SP1 protease cleavage sites. In addition, to examine how the assembly of the chimeric SIV Gag polyproteins is modulated by the FIV sequences that lie directly C-terminal to the CA domain, we constructed two chimeric proviral DNAs: SIV_FIV CA-p1_, in which the FIV spacer peptide p1 was linked to the last seven residues of SIV SP1, and SIV_FIV CA-p1-NC(1-9)_, in which the FIV sequences encoding the entire CA domain as well as p1 and the first nine residues of NC were substituted for the equivalent region in the SIV_SMM-PBj_ genome (Fig. 1). Moreover, the chimeras SIV_FIV CA(NTD)_ and SIV_FIV CA(CTD)_ were designed to study the functional homology between the SIV and FIV CA N-terminal and C-terminal domains, respectively (Fig. 1).

### Assembly phenotype of the SIV chimera carrying the FIV CA domain

**Figure 2:**
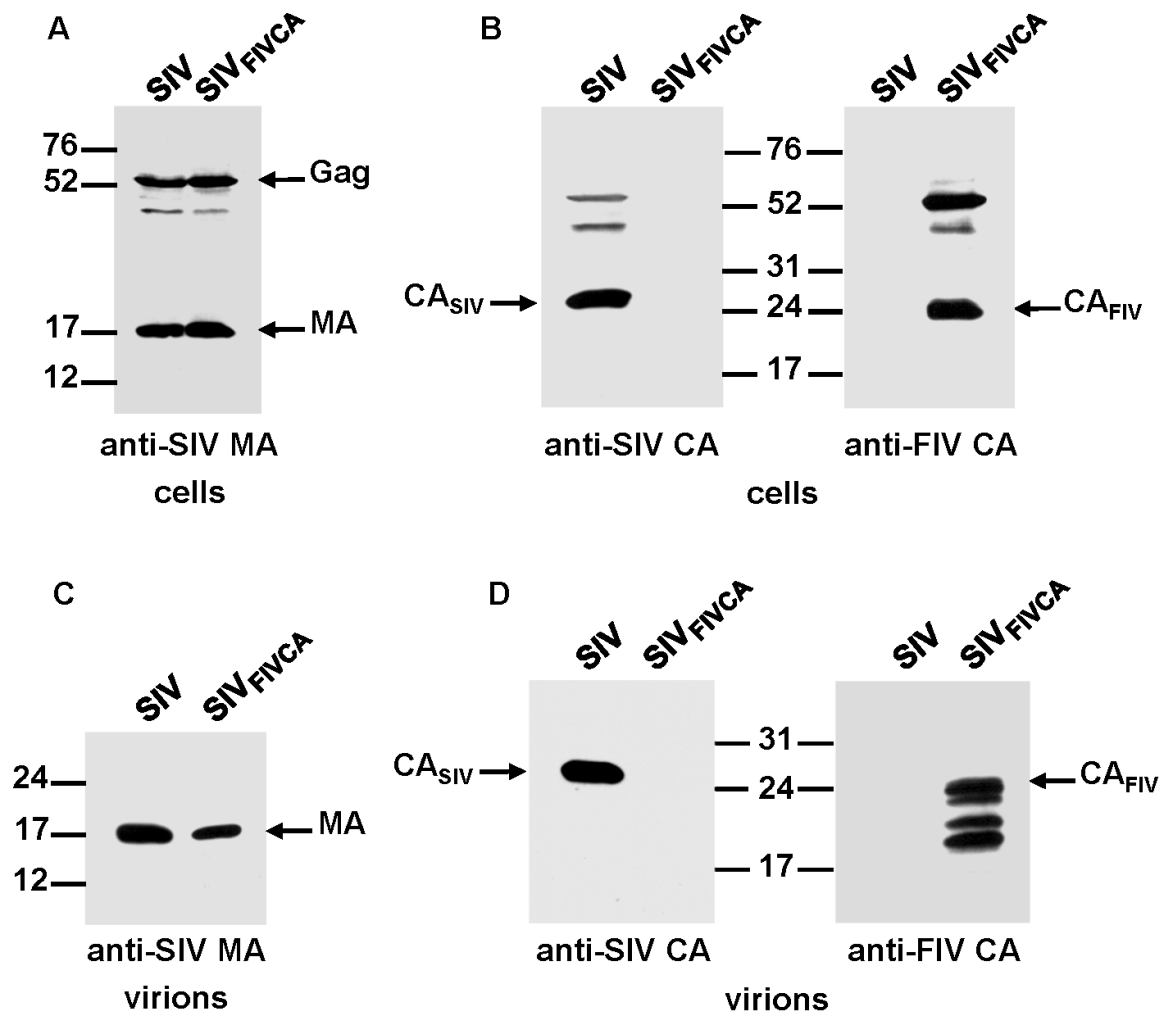
Assembly phenotype of the chimeric SIV_FIV CA_ virus. 293T cells were transfected in parallel with the wild-type SIV_SMM-PBj_ and SIV_FIV CA_ proviral DNAs. At 48 h post-transfection, cell and virion lysates were resolved on SDS-polyacrylamide gels, transferred to nitrocellulose membranes and detected with antibodies specific for the SIV MA (A and C) and for the SIV and FIV CA proteins (B and D). The positions of the wild-type and chimeric Gag proteins as well as those of the MA and CA proteins are shown. Numbers refer to the positions of the molecular weight standards (in kDa).

To determine whether the FIV CA is able to functionally replace the equivalent SIV domain and confer assembly competence to the chimeric SIV Gag, we transfected the wild-type SIV and chimeric SIV_FIV CA_ proviral DNAs into 293T cells. Analysis of the cell and virion lysates by Western blotting using an antiserum directed against the SIV MA showed that the chimeric SIV_FIV CA_ Gag polyprotein was expressed and processed at wild-type levels and that it assembled into virions (Fig. 2A and 2C). However, probing for the SIV and FIV CA proteins revealed that the chimeric SIV_FIV CA_ particles contained, besides the mature FIV CA protein, additional low-molecular-weight bands derived from the FIV CA (Fig. 2B and 2D), which suggests that maturation of the chimeric SIV_FIV CA_ virions is accompanied by certain degree of instability of the FIV CA domain.

### Particle production by the SIV chimera expressing the FIV CA-p1 domain

**Figure 3:**
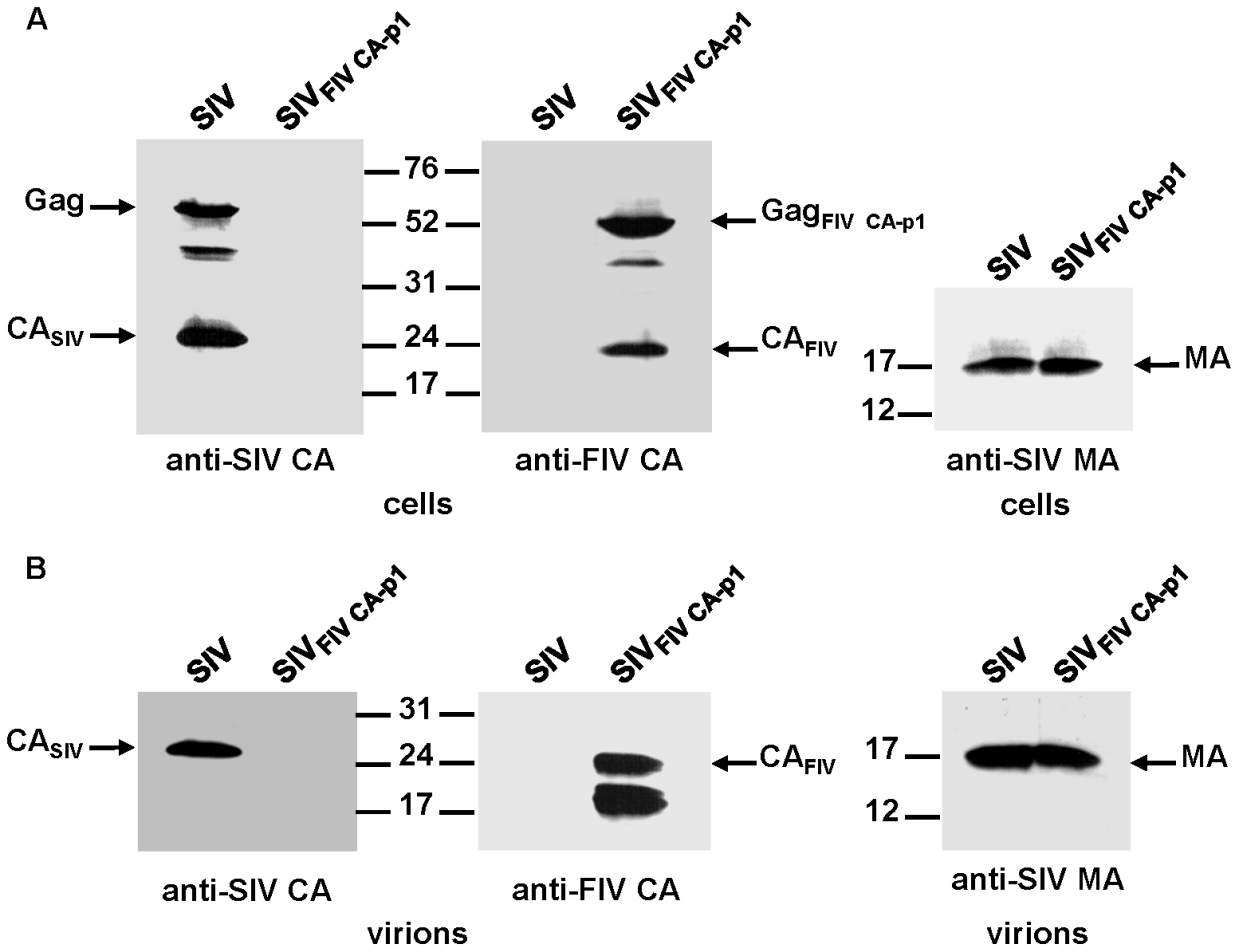
Assembly phenotype of the SIV_FIV CA-p1_ chimera. 293T cells were transfected in parallel with wild-type SIV_SMM-PBj_ and SIV_FIV CA-p1_ proviral DNAs. Protein blots of cell (A) and virion (B) lysates were probed with antibodies specific for the SIV MA, SIV CA, and FIV CA proteins. The positions of the wild-type and chimeric Gag precursors, as well as those of the mature SIV MA, SIV CA, and FIV CA proteins are shown. Numbers indicate the positions of the molecular weight standards (in kDa).

Based on the results described above, we next asked whether the inclusion of the FIV p1 spacer peptide in place of the first 10 amino acids of SP1 could reverse the FIV CA instability observed for the mature chimeric SIV_FIV CA_ particles. Immunoblotting of both cell lysates (Fig. 3A) and virion fractions (Fig. 3B) of transfected cells with the anti-FIV CA MAb indicated that the chimeric SIV_FIV CA-p1_ virions display additional FIV CA-derived bands of a molecular mass lower than 24 kDa similar to the protein pattern of the chimeric SIV_FIV CA_ virions.

### Assembly of the SIV_FIV CA-p1-NC(1-9)_ chimera

**Figure 4:**
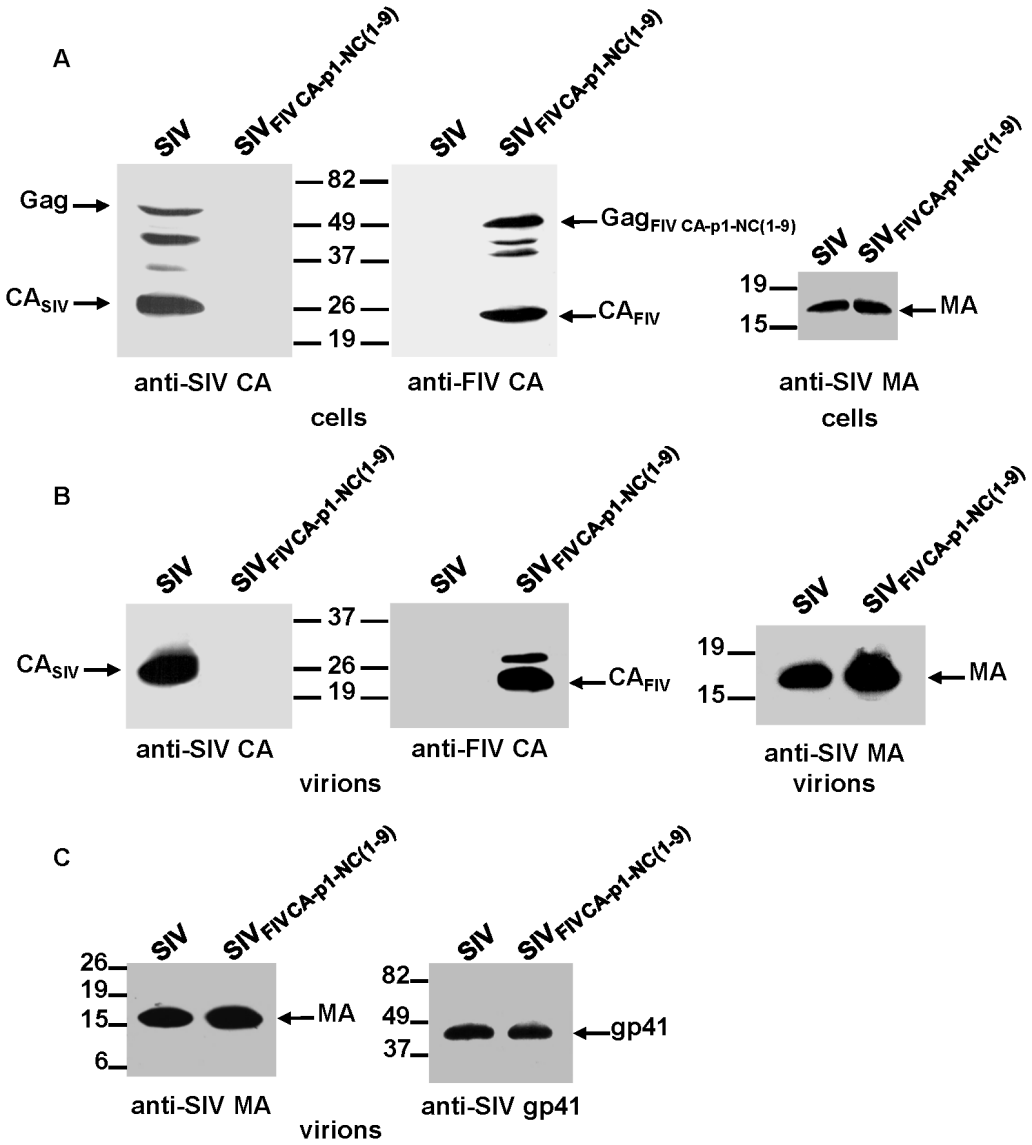
Analysis of particle production and Env incorporation into virions for SIV_FIV CA-p1-NC(1–9)_. 293T cells were transfected in parallel with wild-type SIV_SMM-PBj_ and SIV_FIV CA-p1-NC(1–9)_ proviral DNAs. Cell- (A) and virion-associated (B) proteins were detected by Western blotting with antibodies directed against the SIV MA, the SIV CA, and the FIV CA proteins. (C) Env incorporation into the wild-type SIV_SMM-PBj_ and chimeric SIV_FIV CA-p1-NC(1–9)_ virions was determined by probing with an anti-SIV gp41 MAb (right panel). Virions were normalized for their MA content (left panel). The positions of the wild-type and chimeric Gag precursors, as well as those of the mature SIV MA, SIV CA, FIV CA and SIV gp41 proteins are shown. Numbers indicate the positions of the molecular weight standards (in kDa).

We then examined the assembly competence of a chimeric SIV expressing the FIV Gag region comprising the CA domain, p1, and the NC sequences upstream of the first zinc-finger motif (SIV_FIV_ _CA-p1-NC(1–9)_; Fig. 1). The proviral DNAs for wild-type SIV_SMM-PBj_ and the SIV_FIV CA-p1-NC(1–9)_ chimera were transfected into 293T cells and both the cell and virion lysates were then assayed for the presence of viral proteins by Western blotting using the anti-SIV MA serum or the MAbs specific for either the SIV or FIV CA proteins (Fig. 4). The SIV_FIV CA-p1-NC(1–9)_ chimeric virus exhibited an assembly-competent phenotype similar to that of the SIV_FIV CA_ and SIV_FIV CA-p1_ chimeras; however, in contrast to the latter chimeric viruses, the SIV Gag_FIV CA-p1-NC(1–9)_ polyprotein assembled into virions containing a mature and stable FIV CA protein (Fig. 4B). In addition, a Gag intermediate of higher molecular mass, most likely corresponding to FIV CA-p1-NC, was also detected in these chimeric particles (Fig. 4B).

### Infectivity, reverse transcriptase activity and RNA content of the SIV_FIV CA-p1-NC(1–9)_ virions

**Figure 5:**
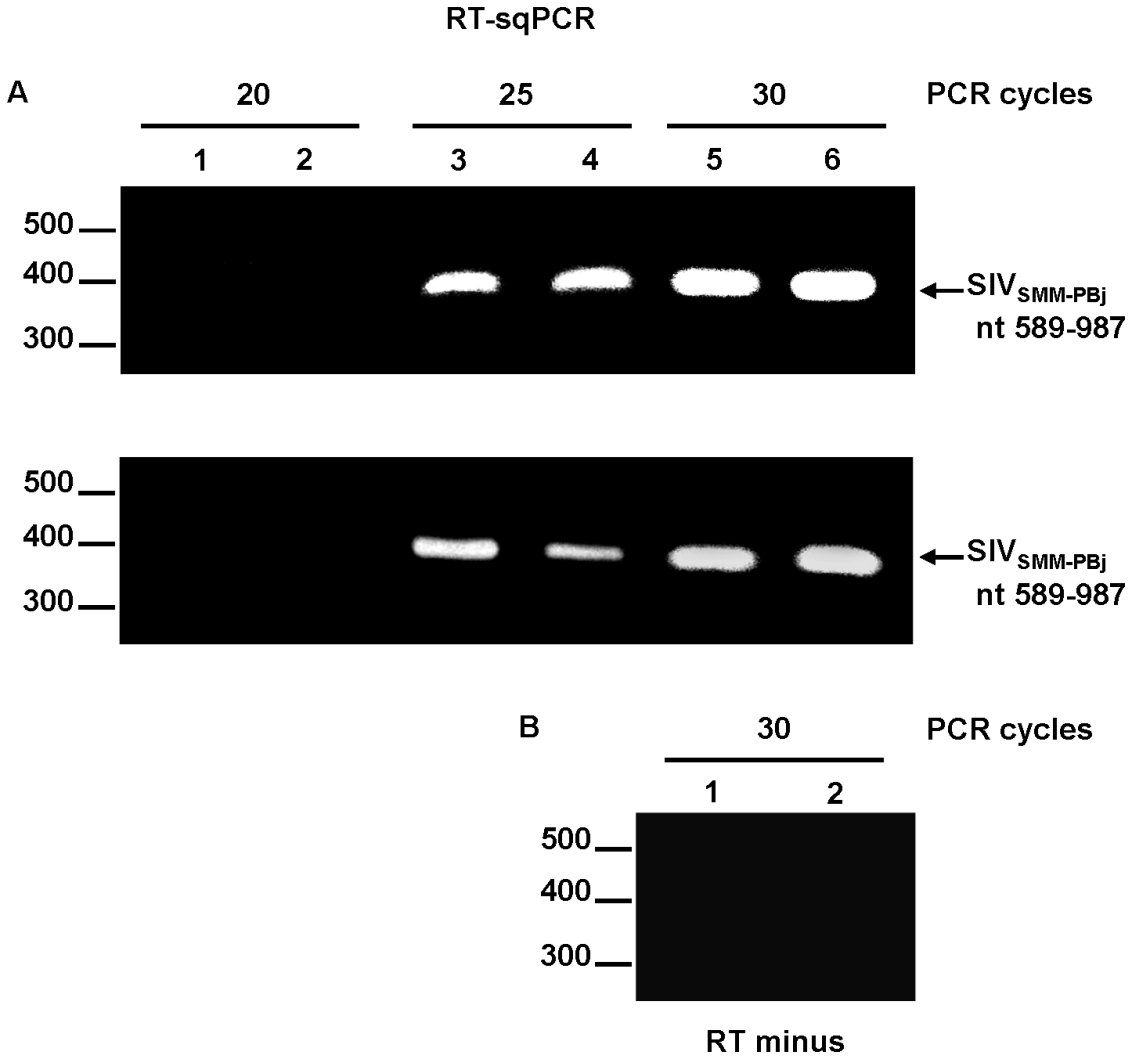
Analysis of the genomic RNA content in the chimeric SIV_FIV CA-p1-NC(1–9)_ virions by RT-sqPCR. (A) Upper panel: Equal aliquots of first-strand cDNAs prepared from genomic RNA extracted from equal amounts of wild-type SIV (lanes 1, 3, and 5) and SIV_FIV CA-p1-NC(1–9)_ virions (lanes 2, 4, and 6) were used as templates in PCR reactions involving 20, 25, or 30 cycles for the amplification of the SIV genomic region encompassing nt 589–987. Lower panel: In parallel, the same round of PCR cycles as above was carried out using one-half volume of the cDNA product from SIV_FIV CA-p1-NC(1–9)_ virions (lanes 2, 4, and 6) with respect to that from wild-type virions (lanes 1, 3, and 5). (B) Parallel reactions (30 PCR cycles) for genomic RNAs extracted from wild-type SIV (lane 1) or SIV_FIV CA-p1-NC(1–9)_ (lane 2) virions performed without the addition of RT (RT minus) demonstrates the absence of plasmid DNA contamination. The reaction products were separated by agarose gel electrophoresis and visualized by ethidium bromide staining. Numbers on the left refer to the positions of the DNA molecular weight markers (in base pairs), whereas the viral genomic RNA region amplified by RT-PCR using the SIV-specific 5′and 3′primers is indicated on the right.

Given the stability of the FIV CA protein in the virions produced by SIV_FIV CA-p1-NC(1–9)_, we decided to investigate whether this chimeric virus was infectious. When the culture supernatants from transfected cells were used to assess virus infectivity in TZM-bl cells, we found that the chimeric SIV_FIV CA-p1-NC(1–9)_ virus had only 3.0±0.9% of wild-type infectivity (average of five independent experiments ± the standard deviation). It should be mentioned that the TZM-bl cells derive from the HeLa cell line which is only partially restrictive to FIV replication [58]. The reduced infectivity of SIV_FIV CA-p1-NC(1–9)_ could not be attributed to impaired Env glycoprotein incorporation since probing of the virion samples with an anti-gp41 MAb showed that the chimeric particles exhibited gp41 levels similar to those of wild-type SIV (Fig. 4C). In addition, pseudotyping of the chimeric virus with the vesicular stomatitis virus glycoprotein (VSV-G) did not increase virus infectivity in the indicator TZM-bl cells (data not shown). These results prompted us to analyze whether the low infectivity exhibited by the SIV_FIV CA-p1-NC(1–9)_ virus was due to reduced reverse transcriptase (RT) activity of the chimeric virons and/or to a defect in genomic RNA packaging. After normalizing for the amount of SIV MA protein in the samples, virion-associated RT was measured using an enzymatic assay as described in Materials and Methods. No significant defect in virion-associated RT activity was observed for the SIV_FIV CA-p1-NC(1–9)_ virions as compared to the wild-type values (15.0±0.2 ng RT/ml for the chimera versus 16.0±0.3 ng RT/ml for wild-type SIV; average of three independent experiments ± the standard deviation). We next evaluated whether the FIV Gag-derived sequences affected the RNA packaging efficiency of the SIV_FIV CA-p1-NC(1–9)_ virions. The presence of genomic RNA in viral particles was determined by using reverse transcription coupled to semiquantitative polymerase chain reaction (RT-sqPCR) for the amplification of the SIV sequence spanning the packaging signal ψ and the 5′end of the MA-coding region. The resulting DNA products were then quantitated by densitometry of the stained agarose gels. We have previously applied this strategy to quantitate the RNA content in *in vitro* assembled FIV Gag particles [8]. Wild-type and chimeric virion samples were first normalized for SIV MA protein levels so as to compare equal amounts of viral particles with respect to RNA content. As shown in Fig. 5A (upper panel), after 25 or 30 PCR cycles, similar amounts of the amplified DNA product were detected from the wild-type SIV and chimera samples. As control, when the RT was omitted form the reactions no DNA products were obtained (Fig. 5B). To confirm that under our experimental conditions exponential amplification occurred during 25 cycles, one-half volume of the first-strand cDNA from SIV_FIV CA-p1-NC(1–9)_ RNA with respect to that of wild-type virions was subjected to PCR. Using this amount of cDNA from the chimera in 25-cycle reactions we found that the levels of the corresponding PCR product represented 59±6% of the wild-type value (Fig. 5A, lower panel). By contrast, 30 PCR cycles yielded comparable amounts of the wild-type and chimeric PCR products (Fig. 5A, lower panel), indicating that the plateau phase of the reaction is indeed reached in the 30-cycle reactions. Therefore, based on the fact that similar amounts of PCR product are obtained for both the wild-type SIV and chimera samples during 25 cycles of exponential amplification (Fig. 5A, upper panel), we conclude that SIV_FIV CA-p1-NC(1–9)_ virions package genomic RNA as efficiently as wild-type SIV particles.

### Analysis of the replication capacity of the SIV_FIV CA-p1-NC(1–9)_ chimera in non-restrictive 293T cells

**Figure 6:**
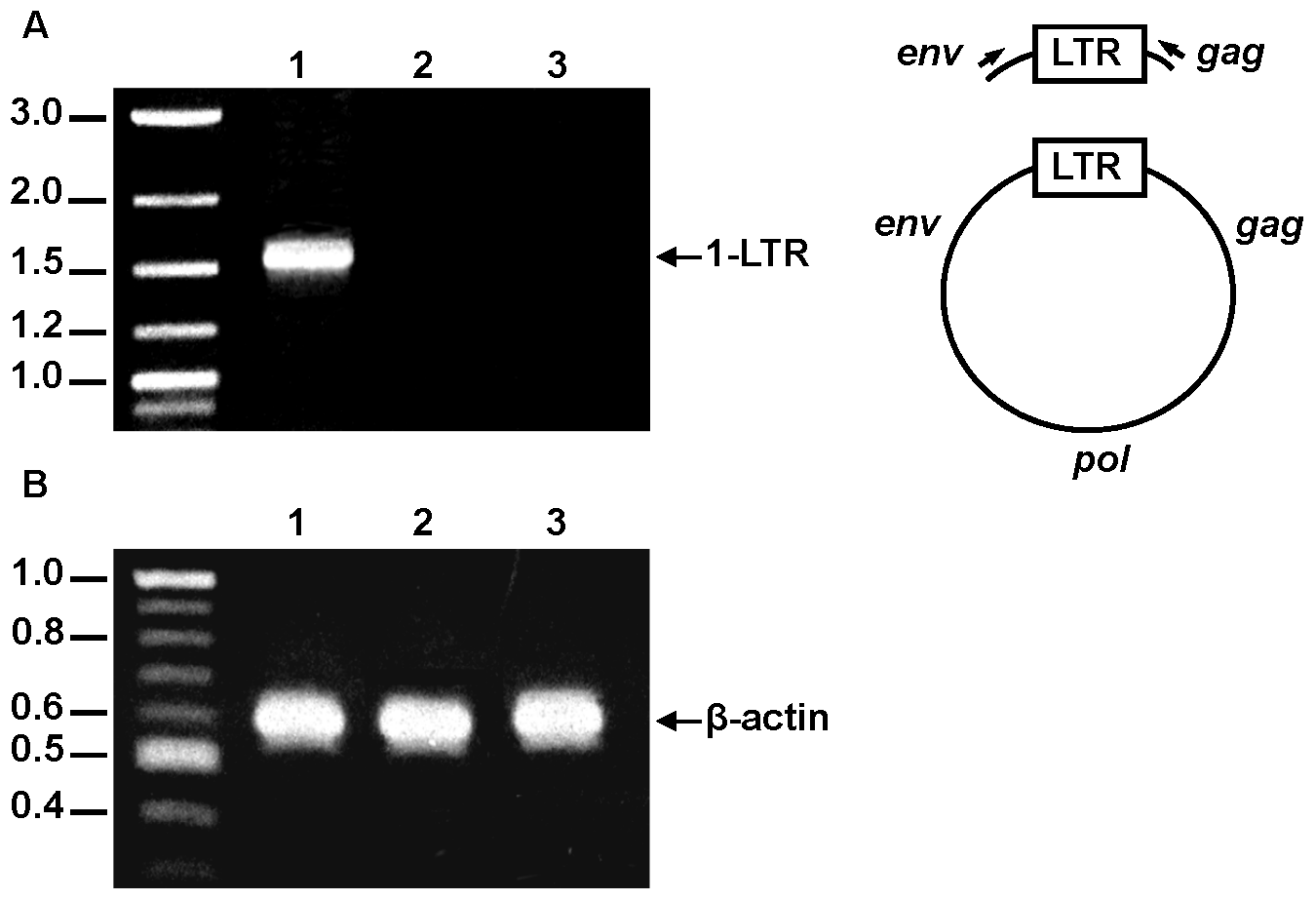
Analysis of the replication capacity of the SIV_FIV CA-p1-NC(1–9)_ chimera in permissive 293T cells by detecting the nuclear circular forms of unintegrated viral DNA. (A) Left panel: Detection of late RT products at 48 h post-infection of 293T cells with VSV-G-pseudotyped wild-type SIV_SMM-PBj_ (lane 1), VSV-G-pseudotyped SIV_FIV CA-p1-NC(1–9)_ (lane 2), or *env*-minus SIV_SMM-PBj_ (lane 3). Right panel: Schematic representation of the PCR product resulting from the 1-LTR circle using the 5′*env*- and 3′ *gag*-specific primers (denoted as arrows). (B) To control for the amount of total DNA in each sample, a human β-actin gene region was amplified from an aliquot of total DNA from cells infected with VSV-G-pseudotyped wild-type SIV_SMM-PBj_ (lane 1), VSV-G-pseudotyped SIV_FIV CA-p1-NC(1–9)_ (lane 2), or *env*-minus SIV_SMM-PBj_ (lane 3). The PCR amplifications were performed as explained in Materials and Methods. The reaction products were separated by agarose gel electrophoresis and visualized by ethidium bromide staining. Numbers indicate the positions of the DNA molecular weight markers (in kb).

Given that our data showed that the low infectivity of the chimeric SIV_FIV CA-p1-NC(1–9)_ virions was not due to an impairment in Env incorporation, defects in virion-associated RT or genomic RNA packaging, we aimed to identify the steps in the viral life cycle following virus entry that were affected by the presence of the FIV CA-p1-NC_(1–9)_ sequences within the chimeric SIV genome. We therefore examined whether the SIV_FIV CA-p1-NC(1–9)_ chimera is able to undergo reverse transcription by performing single-round infections of 293T cells with VSV-G-pseudotyped viruses. To exclude the possibility that the tripartite motif protein TRIM5-α may mediate restriction of the chimeric virus through targeting of the FIV CA-derived sequences, we chose the 293T cell line for this experiment since it has proven to be notably permissive for FIV [58] and SIV [59] vectors. The detection of the circular forms of unintegrated viral DNA serves as criterion of import of full-length viral DNA into the nucleus and, therefore, of a productive viral infection [60]–[62]. Circular low-molecular-weight DNA from wild-type SIV- and chimeric SIV_FIV CA-p1-NC(1–9)_-infected cells was isolated and PCR amplified with primers specific for the late circular DNA forms as described in Materials and Methods. As shown in Fig. 6A, while the 1-LTR-circle species was present in cells infected with wild-type SIV, this DNA form was not detected in SIV_FIV CA-p1-NC(1–9)_-infected cells. The absence of the LTR-derived PCR product in the latter case is not due to differences in the total number of cells in each sample, since amplification of the human β-actin gene from total cellular DNA of both samples yielded equivalent amounts of the expected PCR product (Fig. 6B). DNA sequencing confirmed that the PCR product obtained from wild-type SIV-infected cells corresponds to the 1-LTR circular form (data not shown). Moreover, it is well established that in HIV-1-, SIV-, and FIV-infected cells the 2-LTR form is less abundant than the 1-LTR circle [60]–[62], which is in agreement with our results.

### Replacement in SIV Gag of the CA N-terminal or C-terminal domains by the equivalent regions of FIV CA

**Figure 7:**
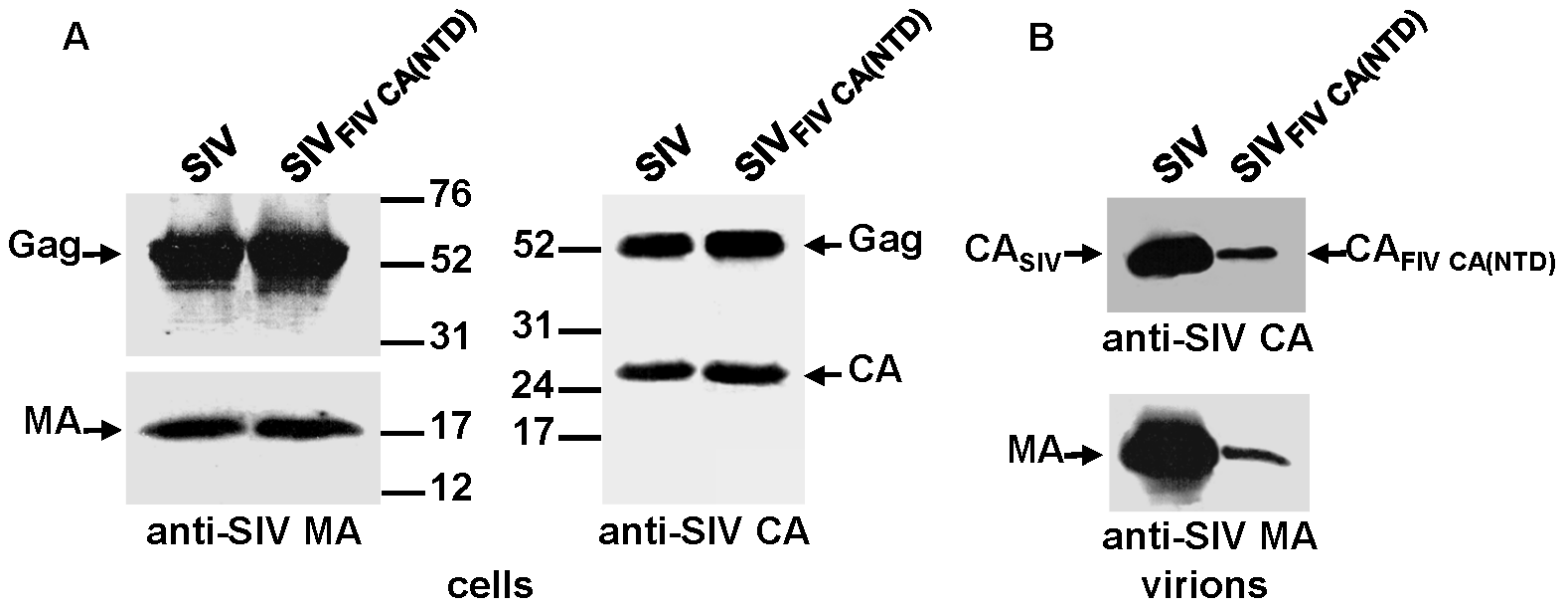
Effect of the replacement of the SIV CA-NTD by its FIV counterpart on Gag assembly. 293T cells were transfected in parallel with the wild-type SIV_SMM-PBj_ and SIV_FIV CA(NTD)_ proviral DNAs. At 48 h post-transfection, cell (A) and virion (B) lysates were resolved on SDS-polyacrylamide gels, transferred to nitrocellulose membranes and detected with antibodies specific for the SIV MA and CA proteins. The relative mobilities of the wild-type and chimeric Gag and CA proteins are shown, as well as that of the SIV MA. Numbers refer to the positions of the molecular weight standards (in kDa).

**Figure 8:**
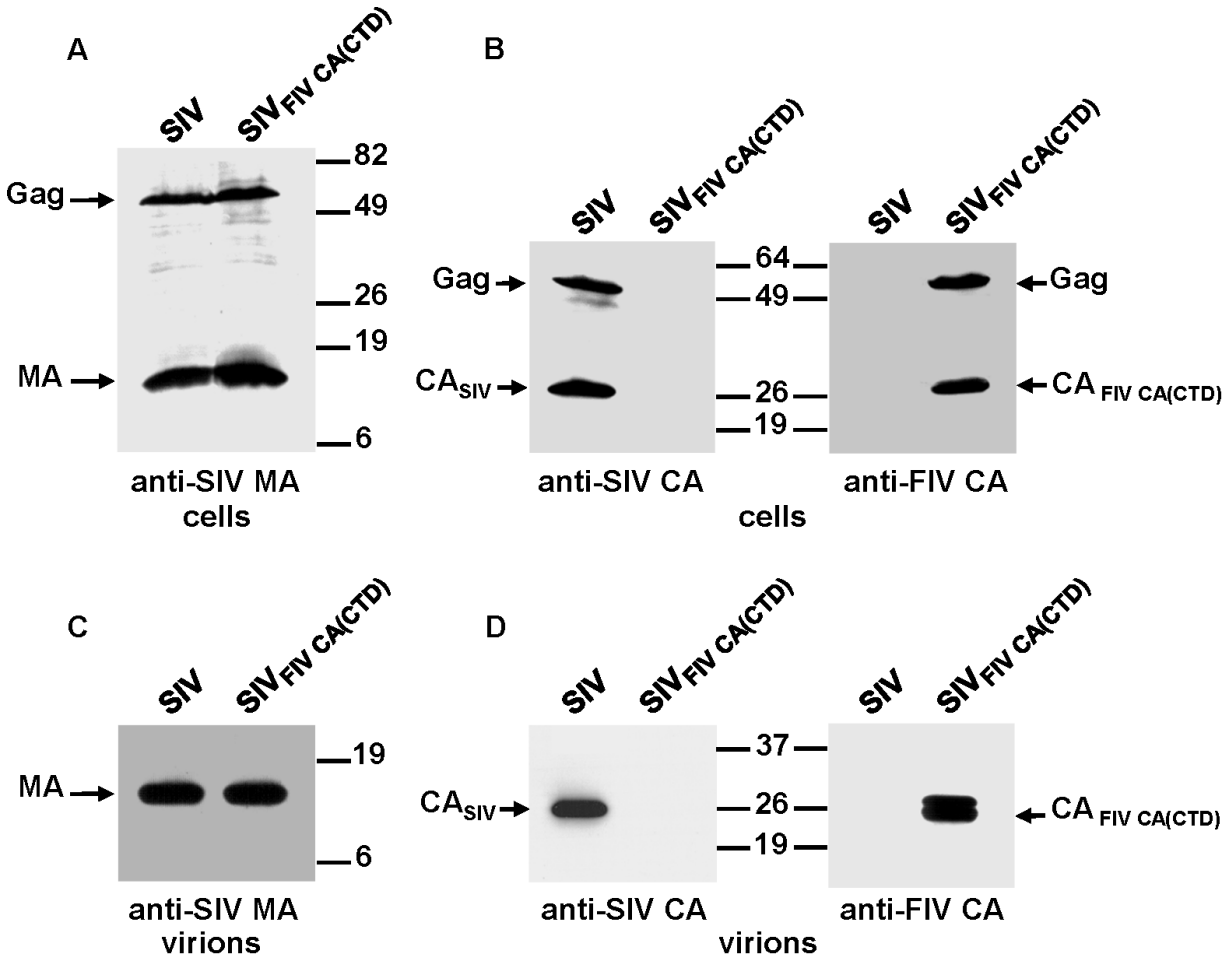
Effect on Gag assembly of the replacement of the SIV CA-CTD by its FIV counterpart. 293T cells were transfected in parallel with the wild-type SIV_SMM-PBj_ and SIV_FIV CA(CTD)_ proviral DNAs. Protein blots of cell (A and B) and virion (C and D) lysates were probed with antibodies specific for the SIV MA (A and C), SIV CA (B and D), and FIV CA (B and D) proteins. The relative mobilities of the wild-type and chimeric Gag and CA proteins are shown, as well as that of the SIV MA protein. Numbers refer to the positions of the molecular weight standards (in kDa).

To further investigate the relationship between the SIV and FIV CA proteins, we next asked whether either the FIV CA-NTD or the CA-CTD was capable of functionally replacing its cognate SIV CA domain. To this end, we examined the assembly phenotype of the chimeric proviruses SIV_FIV CA(NTD)_ and SIV_FIV CA(CTD)_. 293T cells were transfected in parallel with the wild-type or chimeric proviral DNAs and the resulting cell and virions lysates were analyzed by Western blotting with the anti-SIV MA and either the anti-SIV CA or anti-FIV CA antibodies (Fig. 7 and Fig. 8). The steady-state expression levels and processing patterns of both chimeric Gag polyproteins were similar to those of wild-type SIV Gag (Fig. 7A, 8A and 8B). However, these chimeric viruses exhibited markedly distinct assembly phenotypes: while the substitution of the FIV CA-NTD for the equivalent region in the SIV CA was completely detrimental to virion assembly (Fig. 7B), the FIV CA-CTD proved to be able to drive the assembly of the chimeric SIV Gag_FIV CA(CTD)_ precursor into particles (Fig. 8C). It should be mentioned that a chimeric SIV carrying the first 13 residues of the SIV CA joined to amino acids 14–142 of the FIV CA-NTD exhibited the same assembly-incompetent phenotype as the chimera SIV_FIV CA(NTD)_ (data not shown). Of note, the viral particles produced by SIV_FIV CA(CTD)_ contained, besides the expected mature chimeric SIV CA_FIV CA(CTD)_ protein a Gag-derived processing product that, based on its molecular mass and antibody reactivity, corresponds to the chimeric SIV CA_FIVCA(CTD)_-SP1 intermediate (Fig. 8D). Similar to the case of SIV_FIV CA-p1-NC(1–9)_, we found that the chimeric SIV_FIV CA(CTD)_ virions incorporated the Env glycoprotein at levels comparable to those of wild-type SIV_SMM-PBj_, but were non-infectious in the infectivity assays using the indicator TZM-bl cells or in permissive 293T cells infected with the VSV-G-pseudotyped chimeric virus, as judged by the absence of the viral circular DNA forms in infected cells (data not shown).

### Oligomerization ability of the C-terminal domain of the FIV CA

**Figure 9:**
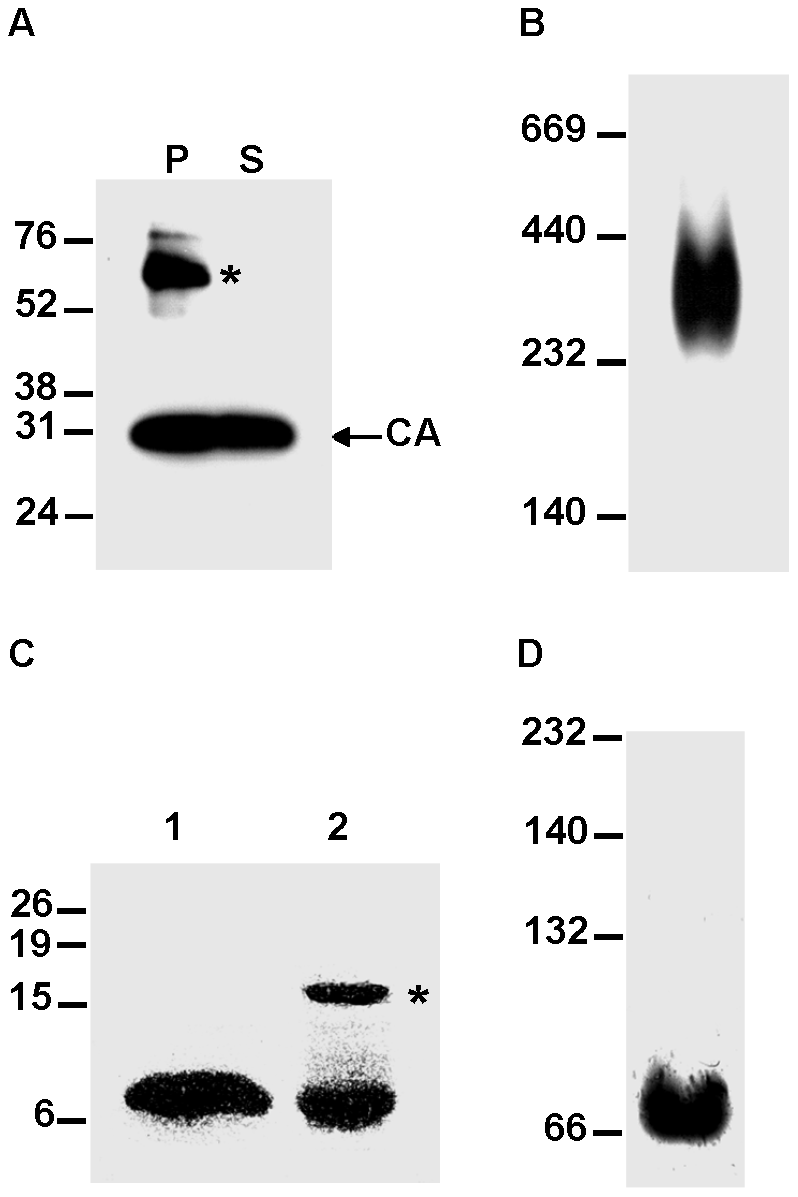
*In vitro* oligomerization ability of the FIV CA and FIV CA-CTD polypeptides. (A) Sedimentation analysis of the *in vitro* assembly reaction for recombinant FIV CA. The purified His-FIV CA protein was incubated under the conditions described in Materials and Methods and the assembly mixture was separated by centrifugation into the pellet (P) and supernatant (S) fractions which were then subjected to SDS-PAGE followed by Western blotting using an anti-FIV CA MAb. The asterisk indicates SDS-resistant CA dimers. (B) To examine the oligomeric arrangement of the FIV CA, an aliquot of the *in vitro* assembly reaction for the recombinant FIV CA protein was analyzed by native gel electrophoresis (5% non-denaturing polyacrylamide gel) followed by Western blotting using an anti-FIV CA antibody. (C) Electrophoretic analysis of the FIV CA-CTD polypeptide under denaturing conditions. The His-tagged FIV CA-CTD protein was purified by affinity chromatography, digested with enterokinase, and run on an SDS-polyacrylamide gel in Laemmli loading buffer with (lane 1) or without SDS (lane 2) followed by staining with Coomassie G-250. The asterisk denotes dimers of the FIV CA-CTD. (D) Assembly reaction for the FIV CA-CTD polypeptide. The recombinant FIV CA-CTD protein was incubated under the same conditions used for the FIV CA and the assembly mixture was then analyzed by Western blotting of native gels using the anti-FIV CA MAb. The migration positions of the molecular mass markers (in kDa) for the denaturing (A and C) and native (B and D) gel electrophoresis are indicated on the left.

Our analysis of the assembly competence of the chimeric SIV Gag polyproteins pointed to a functional plasticity character of the FIV CA-CTD since it had proven to be the minimal FIV CA-derived region capable of replacing its SIV CA counterpart in the context of the entire SIV Gag precursor during virus particle assembly and maturation. To investigate this issue further, we examined whether the FIV CA-CTD is able to oligomerize in solution by performing *in vitro* assembly reactions with recombinant FIV CA-CTD similar to those that we have described for the SIV [9] and FIV Gag polyproteins [8]. In parallel, we analyzed, as control, the products resulting from the *in vitro* assembly reaction of the FIV CA protein. Both the FIV CA and FIV CA-CTD polypeptides were overexpressed in *Escherichia coli* as N-terminally His-tagged fusion proteins and purified by affinity chromatography. The recombinant FIV CA-CTD protein was further treated with enterokinase as described in Materials and Methods to remove the histidine tag. The multimerization ability of the purified FIV CA protein was determined by analyzing the products of the *in vitro* assembly reactions by both sedimentation assays [7] and native gel electrophoresis as we have previously reported [8], [9]. As shown in Fig. 9A, analysis of the pellet and soluble fractions obtained after centrifugation of the assembly reactions showed that a substantial proportion of the recombinant FIV CA protein partitioned in the pellet fraction. In addition, a sodium dodecyl sulfate (SDS)-resistant protein species that might represent FIV CA dimers was detected in the pellet fraction (Fig. 9A). In this regard, it is noteworthy to mention that the SIV CA protein is also capable of forming SDS-resistant dimers [9]. When the *in vitro* assembly reaction of the FIV CA protein was directly analyzed by native gel electrophoresis coupled to Western blotting using the MAb specific for the FIV CA, a high-molecular-mass oligomer of 310–340 kDa was detected (Fig. 9B). This protein species is compatible with the self-assembly of the FIV CA into a multimeric complex. Indeed, our data are in keeping with the results recently reported showing that the FIV CA is mainly dimeric at high protein concentration and that it assembles into higher-order oligomers [63].

To determine whether the CA-CTD is responsible for the ability of the FIV CA to dimerize and thereby form high-molecular-mass complexes, we first evaluated how the addition of SDS to the sample buffer affected the electrophoretic behavior of the purified recombinant FIV CA-CTD polypeptide. As expected, under totally denaturing conditions, the FIV CA-CTD migrated in SDS-polyacrylamide gels with an apparent molecular mass of 7.4 kDa (Fig. 9C, lane 1). Interestingly, when the SDS was omitted from the sample buffer an additional band of 16.2 kDa was consistently detected which, due to its electrophoretic mobility, most likely corresponds to a CA-CTD dimer (Fig. 9C, lane 2). Furthermore, analysis of the *in vitro* assembly reaction of the FIV CA-CTD by native gel electrophoresis revealed that this polypeptide self-assembles into 74.8 kDa oligomers (Fig. 9D). Similar results were obtained when we applied other nondenaturing electrophoresis systems such as the Blue Native [64] and Tris-His-based methods [65] (data not shown). Collectively, these results demonstrate that the FIV CA-CTD oligomerizes *in vitro*, and that the FIV CA-CTD dimers are likely to constitute the basic assembly unit necessary for the formation of the FIV CA multimers that we detect in the *in vitro* assembly reactions.

## Discussion

A distinctive biological feature of the retroviral Gag polyproteins is their ability to drive virion morphogenesis. In particular, the CA domain of Gag is involved not only in the assembly and maturation steps of the viral particles but also in the early stages of the virus life cycle such as virion uncoating and nuclear import of the preintegration complex [37]. Based on the accepted view that all retroviral CA proteins are structurally organized in two different domains, we decided to investigate the functional homology between the CA proteins of two distantly related lentiviruses. We therefore characterized chimeric SIVs in which the *gag* region coding for the CA domain was partially or fully replaced by its equivalent FIV counterpart. Substitution of the FIV CA residues 14 to 222 alone (chimera SIV_FIV CA_) or together with the adjacent p1 peptide (chimera SIV_FIV CA-p1_) for the equivalent region of SIV Gag does not affect the ability of the chimeric viruses to assemble into particles. However, maturation of both chimeric SIV_FIV CA_ and SIV_FIV CA-p1_ virions triggers CA instability, which may reflect that, upon processing of the chimeric Gag, the FIV CA is unable to form a stable core lattice structure. The strategy for the construction of these chimeric viruses was based on the studies performed in HIV-1 which have demonstrated that proteolytic processing at the MA-CA site of the Gag precursor induces the refolding of the first thirteen residues in the mature CA protein into a β-hairpin structure stabilized by a salt bridge between Pro1 and Asp51 [29], [66]. This β-hairpin structure is essential for the generation of mature CA hexamers which in turn is necessary for the formation of mature infectious virions [17], [67]. Of note, the structural study of the equine infectious anemia virus CA has led to the proposal that the function of the maturational refolding in CA is to extend α-helix H1 at the N terminus to enhance the multimerization of the CA-NTD for assembly [68]. In this regard, in both SIV_FIV CA_ and SIV_FIV CA-p1_ chimeras the N-terminal 13 residues of the SIV CA, which share 54% similarity with the equivalent region in the FIV CA, were maintained and joined to the downstream sequences coding for the FIV CA residues 14–222 so as not to affect the formation of a salt bridge between SIV CA Pro1 and FIV CA Asp50 during maturation of the chimeric SIV Gag polyproteins. It should be mentioned that the Asp residue predicted to be involved in the salt bridge with Pro1 lies at position 50 in both SIV and FIV CA proteins. Therefore, the CA instability observed for these chimeric SIV Gag particles is unlikely to result from an impairment in the formation of the Pro1-Asp50 salt bridge. By contrast, the SIV_FIV CA-p1-NC(1–9)_ and SIV_FIV CA(CTD)_ proviral constructs produced virions exhibiting an intact and stable mature CA protein. We demonstrate here that both the SIV_FIV CA-p1-NC(1–9)_ and the SIV_FIV CA(CTD)_ chimeras are able to assemble into virions that incorporate the Env glycoprotein, package wild-type levels of the viral genomic RNA and contain a functional RT. However, these chimeric viruses are non-infectious. When the VSV-G-pseudotyped chimeric viruses were used to infect permissive cells no evidence of the nuclear viral circular DNA forms, which are indicative of productive infection, was obtained. Therefore, it is likely that these assembly-competent chimeric viruses are non-infectious due to a defect at a post-entry step, such as uncoating of the viral core, reverse transcription, or nuclear import of the preintegration complex. Moreover, it has been shown that although only about 50% of the total HIV-1 mature CA protein assembles into the viral core [69], [70], the presence of small amounts of Gag processing intermediates such as CA-SP1 exert a dominant-negative effect on virion maturation and infectivity [71]–[73]. Of note, the murine leukemia virus MA-CA intermediate also acts as a strong trans-dominant inhibitor of virus maturation and infectivity [74]. Therefore, given that the SIV_FIV CA-p1-NC(1–9)_ and the SIV_FIV CA(CTD)_ virions exhibit, in addition to a stable mature CA protein, the Gag processing intermediates CA-p1-NC and CA-SP1, respectively, it could be speculated that these virion-associated Gag subproducts are responsible for the non-infectious phenotype of these chimeras. In this regard, similar defective phenotypes have been observed for some HIV-1 CA mutants [17], [75]. Our results together with those in HIV-1 highlight the relevance of the CA protein in organizing the structure for viral replication that ensures that the disassembly/uncoating and reverse transcription steps proceed in an ordered and synchronized manner upon viral entry.

Of note, while the FIV CA-CTD is able to provide a protein-protein interface that promotes the assembly of the chimeric SIV Gag_FIV CA(CTD)_ polyprotein, the FIV CA-NTD proved to be functionally incompatible with the rest of the SIV Gag sequences and cannot serve as an assembly platform for the chimeric SIV Gag_FIV CA(NTD)_. The difference in the assembly behavior of these chimeric viruses may be attributed in part to the fact that the CA-CTDs of SIV and FIV share higher sequence homology (43.6% sequence identity; 65.4% sequence similarity) than their respective CA-NTDs (23.6% sequence identity; 45.1% sequence similarity). Moreover, based on the study by Bharat *et al.* on retroviral particles [21], the assembly-defective phenotype of SIV_FIV CA(NTD)_ may be explained by the inability of the FIV CA-NTD to establish the specific intermolecular CA-NTD-CA-NTD interactions required for the formation of the immature chimeric SIV Gag lattice. Furthermore, our results suggest that the defect imposed by the FIV CA-NTD cannot be compensated by the SIV Gag sequences present in the chimera which provide a potentially functional CTD-CTD homodimer interface together with the adjacent SP1 linker region. In short, our data indicate that the FIV CA-NTD cannot functionally replace its SIV counterpart.

It should also be taken into account that the SIV and FIV Gag domains display some differences with respect to their involvement in the protein-protein interactions that drive Gag multimerization. Indeed, we have previously shown that while the SIV MA-CA subdomain and the mature MA and NC proteins interact with SIV Gag [9], their FIV counterparts are unable to associate with wild-type FIV Gag [57]. In addition, it has been reported that, outside the CA region, the different structures that the Gag polyprotein precursor adopts reflect the diversity of the *Retroviridae* family [19].

The phenotypic differences that we found between the chimeric SIV_FIV CA(NTD)_ and SIV_FIV CA(CTD)_ also appear to point to a functional plasticity of the CA-CTD as opposed to the requirement for a quite invariant conformation for the CA-NTD. Interestingly, and in support of this concept, the characterization of a random library of HIV-1 CA mutants showed that the sequences corresponding to the CA-NTD helices are particularly detrimental to virus viability [76]. Intriguingly, our results showing that the chimeric SIV carrying the heterologous FIV CA-NTD is assembly-incompetent contrast with those of a previous study in which it was concluded that the HIV-1 CA-NTD is nonessential for immature particle assembly [77]. Nevertheless, it can be speculated that in that particular HIV-1 Gag mutant other domains in Gag might compensate for the absence of the CA-NTD region and that removal of the entire CA-NTD might be less detrimental to Gag assembly than the presence of a non-functional CA-NTD.

Furthermore, we show here that the FIV CA protein self-assembles *in vitro*, which is in line with the recent report by Serrière *et al.* [63] showing that this protein forms spherical structures. Interestingly, we also demonstrate that the FIV CA-CTD dimerizes in solution and forms high-molecular-weight oligomers. Our data on the intrinsic oligomerization ability of the FIV CA-CTD together with our finding that the chimeric SIV_FIV CA(CTD)_ virus, which only contains the CTD of the FIV CA domain, is assembly-competent provide biological and biochemical evidences for the functional plasticity character of the CA-CTD. This concept is further supported by recent structural studies on the HIV-1 CA protein which have demonstrated the presence of several conformers for the CA-CTD dimer and the dimeric CA protein [33], [78].

In summary, the present work provides novel data about the functional homology between the CA domains of primate and nonprimate lentiviral Gag polyproteins and contribute to our understanding of how the requirements for the assembly of infectious virions have evolved among retroviruses.

## Materials and Methods

### Chimeric proviral DNAs constructs

All the chimeras were generated by replacing different sequences within the CA-SP1-NC-coding region of the SIV_SMM-PBj_ proviral DNA [79] with the equivalent sequences of the molecular clone FIV-14 of the Petaluma isolate [80]. The SIV and FIV CA-coding regions were joined maintaining the distance between residues Pro1 and Asp50 (Asp51 in HIV-1 CA) which have been shown to form a salt bridge in the mature HIV-1 CA protein [67]. Chimeric proviral DNAs were generated by a PCR-based strategy using the Elongase enzyme high-fidelity PCR mix (Life Technologies-Invitrogen). To generate the SIV_FIV CA_ chimera, the amplified DNA fragment corresponding to SIV nucleotides (nt) 601–1272 was ligated to the DNA coding for the FIV CA residues 14 to 222 (nt 1072–1698 of the FIV-14 genome). The resulting fragment was then joined to the SIV region comprising the last six amino acids of the SIV CA, SP1, NC, SP2 and part of the p6 domain in Gag (nt 1905–2190 of SIV_SMM-PBj_). In the case of the SIV_FIV CA-p1_ chimeric fragment, the FIV sequences coding for the FIV CA residues 14–222 and the spacer peptide p1 (nt 1072–1725) were joined to Gly11 of the SP1 sequence in *gag* (nt 1954 of SIV_SMM-PBj_). For the construction of the chimeric proviral DNA SIV_FIV CA(NTD)_, the SIV region corresponding to the long terminal repeat (LTR) and MA (nt 601–1233) was linked to the sequences encoding the FIV CA residues Pro1-Ala142 (FIV-14 nt 1033–1458). The ligation product was reamplified by PCR and then joined to the SIV sequences coding for the SIV CA-CTD (Leu150 to Met230) together with the downstream *gag* region (nt 1681–2190 of SIV_SMM-PBj_). The SIV_FIV CA-p1-NC(1–9)_ chimera was constructed by sequentially ligating the following PCR-amplified DNA fragments: the 633-bp DNA containing the LTR and MA-coding region of SIV_SMM-PBj_ mentioned above, a fragment coding for the entire FIV CA, the spacer peptide p1 and the first 9 amino acids of NC (FIV-14 nt 1033–1752), and an SIV-derived DNA fragment encoding residues 11 to 52 of the NC domain and the downstream *gag* sequences (nt 2005–2190). The SIV_FIV CA(CTD)_ chimera, in which the FIV CA-CTD was substituted for that of SIV in the context of the SIV_SMM-PBj_ genome, was generated by first ligating the SIV sequences corresponding to the LTR, the MA, and the first 151 residues of CA (nt 601–1686) to the FIV region coding for amino acids 145–222 of CA (FIV-14 nt 1465–1698). The resulting DNA fragment was reamplified by PCR and then linked to the DNA fragment comprising the last 6 residues of the SIV CA and the downstream *gag* sequences (nt 1906–2190). All the chimeric fragments were digested with DraIII-Bsu36I and substituted for the corresponding wild-type region in the SIV_SMM-PBj_ proviral DNA genome. The chimeric proviral constructs were first screened by restriction mapping and then by DNA sequencing to confirm that the DNA fragments were joined in the correct reading frame.

### Cell cultures and transfections

Human 293T and TZM-bl cells [81] (obtained from the NIH AIDS Research and Reference Reagent Program) were grown in Dulbecco's modified Eagle's medium supplemented with 10% fetal bovine serum (GIBCO) following standard protocols. Transfection of 293T cells was performed using Lipofectamine 2000 (Life Technologies-Invitrogen) essentially as previously described [79] and harvested 48 h post-transfection. Stocks of VSV-G-pseudotyped viruses were prepared by cotransfecting 293T cells with each of the proviral DNAs and the pcDNA plasmid coding for the VSV-G protein at a 1∶1 mass ratio as we have previously reported [82]. At 48 h post-transfection, the culture supernatants were filtered (0.45-µm-pore-size membranes), the viral stocks were normalized for RT activity, and used in the infectivity assays as explained below.

### Viral protein analysis by Western blot

Transfected 293T cells were washed twice with ice-cold phosphate-buffered saline (PBS) and lysed at 4 °C in lysis buffer (50 mM Tris-HCl [pH 8.0], 150 mM NaCl, 1% Nonidet P-40, 0.1% SDS, 0.5% sodium deoxycholate, 1 mM phenylmethylsulfonyl fluoride, and 10 µg/ml aprotinin). The culture supernatants from the transfected cells were filtered through 0.45-µm-pore-size syringe filters and virions were pelleted from the clarified supernatants by ultracentrifugation (100,000 x*g*, 90 min, 4 °C) through a 20% (w/v in PBS) sucrose cushion as we have previously reported [83]. Cell- and virion-associated proteins were resolved on SDS–12.5% or −15% polyacrylamide gels, blotted onto nitrocellulose membranes, and analyzed by Western blotting coupled with an enhanced chemiluminescence assay (SuperSignal West Pico Chemiluminescent Substrate, Thermo Scientific) as previously described [9]. FIV Gag-related proteins were detected by using the anti-FIV CA MAb PAK3-2C1 obtained through the NIH AIDS Research and Reference Reagent Program. The MAb KK60 used to detect the SIV Gag and CA proteins as well as the anti-SIV gp41 MAb KK41 were obtained from J. Stott and K. Kent through the MRC AIDS Directed Program. For the detection of the SIV MA protein, blots were probed with a mouse anti-SIV MA polyclonal serum obtained in our laboratory [11]. Horseradish peroxidase (HRP)-conjugated anti-mouse immunoglobulin (Promega) was used as secondary antibody.

### RT activity assay

Quantitation of virion-associated RT in cell-free culture supernatants from transfected cells was performed by using a commercial colorimetric RT assay (Roche Applied Science) as we have previously described [82], [83]. Briefly, Triton X-100-lysed virus samples were mixed with incubation buffer containing digoxigenin-labeled nucleotides and a poly(rA).oligo(dT)_15_ template-primer hybrid and incubated for 2 h at 37°C. Newly synthesized DNA was detected by a HRP-conjugated sheep immunoglobulin G fraction specific for digoxigenin and 2,2′-Azino-bis(3-ethylbenzthiazoline-6-sulfonic acid) substrate. The resulting colored reaction signal was measured on a microtiter plate (ELISA) reader at 405 nm (reference wavelength 490 nm). The absorbance of the samples was then correlated to the calibration curve obtained with the recombinant HIV-1 RT enzyme provided in the kit. All assays were performed in duplicate from at least three independent experiments.

### Infectivity assays using the TZM-bl indicator cells

Virus stocks obtained by transfection of 293T cells were normalized for RT activity, and used to infect in duplicate 4×10^4^ TZM-bl cells in 24-well dishes as previously described [79], [84]. Two days postinfection, cells were fixed with PBS buffer containing 1% formaldehyde and 0.2% glutaraldehyde at room temperature for 5 min and then scored for blue foci formation after staining with 5-bromo-4-chloro-3-indolyl-ß-D-galactopyranoside (X-Gal). Virus entry was quantitated as the total number of blue cells per well by first counting the number of blue cells in at least 20 nonoverlapping fields in each of the two wells. The average number of blue cells per field was multiplied by the total number of fields per well, and the result was referred to the number of blue cells obtained with wild-type SIV_SMM-PBj_.

### Viral genomic RNA packaging into virions

Viral genomic RNA was isolated from virions purified by ultracentrifugation of the culture supernatants of transfected 293T cells as described above. Briefly, equal amounts of virions (normalized to MA protein levels) were incubated with 5 U RNase-free DNase I RQ1 (Promega) in a buffer solution containing 50 mM Tris-HCl (pH 8.0), 10 mM MgCl_2_ and 1 mM CaCl_2_ for 1 h at 37°C followed by treatment with proteinase K (50 µg/ml; 1 h at 37°C) in the presence of 0.5% SDS. After heat inactivation, the mixtures were deproteinized by two phenol: chloroform extractions, and the RNA was then concentrated by ethanol precipitation after adding 10 µg yeast tRNA as carrier. Duplicate samples of the extracted viral genomic RNAs were used as template for RT-sqPCR based on the protocol that we have previously described [8]. The RT reaction was performed using the SuperScript II RT (Life Technologies-Invitrogen) and an antisense oligonucleotide that hybridizes to SIV_SMM-PBj_ nt 967–987, whereas the subsequent PCR step (20, 25, and 30 amplification cycles of 94°C for 30 sec, 56°C for 30 sec, and 72°C for 60 sec) was carried out using a 5′ primer that corresponds to nt 589–611 in the SIV_SMM-PBj_ viral genome and the 3′ oligonucleotide used for the first RT reaction step. Initial RT-PCR pilot experiments were carried out so as to determine the number of cycles necessary to attain the exponential phase. Control reactions without RT were systematically performed with a similar amount of the extracted RNAs to rule out DNA contamination. The RT-PCR products were analyzed by electrophoresis on 2% agarose gels and quantitated by densitometry of the ethidium bromide-stained gels [8].

### Viral replication assay in 293T cells

For the analysis of virus replication in non-restrictive cells, 293T cells were infected with volume-adjusted supernatants containing equivalent RT activity and SIV MA protein levels of VSV-G-pseudotyped viruses (previously treated with DNase I at 25 °C for 20 min to remove potentially contaminating plasmid DNA). After an incubation period of 2–3 h at 37 °C in 5% CO_2_ in the presence of 20 µg/ml DEAE-dextran, infected 293T cells were pelleted, washed twice with PBS to remove residual virus and resuspended in fresh medium. As control, 293T cells were incubated in parallel with an *env*-minus SIV_SMM-PBj_ virus stock. Forty-eight hours post-infection, cells were split into 2–3×10^6^- aliquots, and the cell pellets were kept frozen at −80 °C until used for the isolation of either total cellular DNA or low-molecular-weight DNA as explained in the following section.

### DNA isolation and detection of viral replication intermediates (1-LTR circular form)

Total cellular DNA was purified using the illustra blood genomicPrep Mini Spin Kit (GE Life Sciences) as recommended by the manufacturer. The amplification reactions specific for the β-actin gene, included as an internal control for DNA quantitation and PCR efficiency, were carried out using 300 ng genomic DNA in 50-µl reactions containing 1×Q5 Reaction Buffer (New England Biolabs), 200 µM dNTPs, 0.5 µM each primer (5′-β-actin, 5′-CATGTGCAAGGCCGGCTTCGC-3′ and 3′-β-actin, 5′-CCTTAATGTCACGCACGATTTCC-3′ that target the first and third exon of the human β-actin gene, respectively), and 1 U Q5 High-Fidelity DNA Polymerase (New England Biolabs). After an initial incubation step at 95 °C for 5 min, 25 amplification cycles were performed consisting of 20 s at 95 °C, 30 s at 55 °C and 25 s at 72 °C followed by a final elongation step at 72 °C for 2 min. The procedure for the detection of circular forms of unintegrated viral DNA was based on the strategy described by Cara *et al.* [61]. Low-molecular-weight DNA was isolated from the cell pellets employing the QIAprep Spin miniprep Kit (QIAGEN) and then used as template in amplification reactions. One-LTR circles were detected using the *gag*-reverse primer (5′-CTCCCACTCTCCTACTCTTTTCTC-3′; nt 828-805 of SIV_SMM-PBj_) and the *env*-forward primer (5′-TGGCTATTGAGGAACTGCC-3′; nt 8763–8781 of SIV_SMM-PBj_). PCR was carried out with the Q5 DNA Polymerase as described above using 5 µl of the isolated low-molecular-weight DNA as template and the following PCR parameters: 35 cycles of 95 °C for 20 s, 55 °C for 30 s, 72 °C for 60 s with a final elongation step of 2 min at 72 °C. The PCR products were then analyzed by agarose gel electrophoresis and visualized by ethidium bromide staining. In the case of the amplification reactions for the 1-LTR circular forms, the PCR products were first mapped by restriction endonuclease digestion, then cloned into the EcoRV site of pcDNA3.1, and sequenced.

### Cloning, expression and purification of recombinant FIV CA-derived proteins

The regions coding for the FIV CA (nt 1033–1698 of FIV-14) and the FIV CA-CTD (amino acids Ala137 to Leu222; nt 1441–1698 of FIV-14) were PCR amplified and cloned into the SalI-NotI or NcoI-NotI sites of pET-30b(+), respectively, so as to express the corresponding polypeptides with an N-terminal six-histidine tag. Both polypeptides were produced in *Escherichia coli* strain BL21(DE3) by induction with 1 mM isopropyl β-D-1-thiogalactopyranoside. After 4 h-incubation at 37 °C, the bacteria were pelleted, lysed in ice-cold phosphate buffer (100 mM sodium phosphate [pH 8.0], 300 mM NaCl, 1 mM imidazole and protease inhibitor cocktail [Roche Applied Science]), and incubated with 1 mg/ml lysozyme for 30 min on ice. After treatment with DNase I (5 µg/ml), the bacterial lysates were sonicated, and the His-tagged polypeptides were purified from the clarified protein extracts by affinity chromatography using a nickel-nitrilotriacetic acid resin (Ni-NTA kit; QIAGEN) as we have previously described [8], [9], [11]. Removal of the affinity tag from the His-tagged FIV CA-CTD was performed by incubating the recombinant protein at 23 °C for 16 h with 0.006% (w/w) enterokinase (New England Biolabs), followed by removal of the protease by immunoaffinity (Enterokinase removal kit, SIGMA-ALDRICH) and a second round of nickel chelate chromatography. The protein preparations were finally dialyzed against 50 mM sodium phosphate (pH 7.2). A purity of more than 90% of all protein preparations was confirmed by Coomassie Blue staining of polyacrylamide gels. Protein concentrations were estimated by comparison to known amounts of standard bovine serum albumin on SDS-polyacrylamide gels stained with Coomassie G-250 SimplyBlue Safe Stain (Invitrogen-Life Technologies). Quantitation of the amount of protein on gels was performed by densitometry as we have previously described [8], [9], [11].

### *In vitro* assembly reactions

*In vitro* assembly reactions were performed essentially as we have described previously [8], [9]. The purified FIV CA and FIV CA-CTD proteins (1 µg/µl) were incubated in a buffer solution containing 50 mM Tris-HCl (pH 8.0), 1 M NaCl, 5 mM dithiothreitol during 16 h at 8 °C. The assembly reactions were either directly loaded on nondenaturing polyacrylamide gels as described below or analyzed by sedimentation assays [7]–[9]. In the latter case, the assembly reactions were centrifuged for 1 h in an Eppendorf microcentrifuge at 16,000 x*g* at 4 °C to separate the particulate assembled structures from the unassembled molecules. Supernatant and pellet fractions were resolved by SDS-polyacrylamide gel electrophoresis (SDS-PAGE) and proteins were visualized by immunoblotting with the anti-FIV CA MAb, or by Coomassie blue staining.

### Native electrophoresis analyses of recombinant FIV CA-derived proteins

The oligomeric state of the FIV CA and FIV CA-CTD polypeptides was examined by electrophoresis on 5% nondenaturing Laemmli polyacrylamide gels as we have recently described [9] followed by Coomassie blue staining or Western blotting using the MAb specific for the FIV CA. The molecular masses of the multimeric complexes formed by the FIV CA and FIV CA-CTD were estimated based on the relative mobilities of the molecular weight standards for native electrophoresis (High Molecular Weight Calibration Kit for Native Electrophoresis, GE Life Sciences).
